# Human BCL-G regulates secretion of inflammatory chemokines but is dispensable for induction of apoptosis by IFN-γ and TNF-α in intestinal epithelial cells

**DOI:** 10.1038/s41419-020-2263-0

**Published:** 2020-01-27

**Authors:** Jerzy A. Woznicki, Peter Flood, Milan Bustamante-Garrido, Panagiota Stamou, Gerry Moloney, Aine Fanning, Syed Akbar Zulquernain, Jane McCarthy, Fergus Shanahan, Silvia Melgar, Ken Nally

**Affiliations:** 1 APC Microbiome Ireland, University College Cork, Cork, Ireland; 2 Department of Medicine, University College Cork, Cork, Ireland; 3 Department of Gastroenterology, Mercy University Hospital, Cork, Ireland; 4 School of Biochemistry & Cell Biology, University College Cork, Cork, Ireland

**Keywords:** Apoptosis, Cell death and immune response, Interferons, Tumour-necrosis factors, Mucosal immunology

## Abstract

Proteins of the BCL-2 family are evolutionarily conserved modulators of apoptosis that function as sensors of cellular integrity. Over the past three decades multiple BCL-2 family members have been identified, many of which are now fully incorporated into regulatory networks governing the mitochondrial apoptotic pathway. For some, however, an exact role in cell death signalling remains unclear. One such ‘orphan’ BCL-2 family member is BCL-G (or BCL2L14). In this study we analysed gastrointestinal expression of human *BCL-G* in health and disease states, and investigated its contribution to inflammation-induced tissue damage by exposing intestinal epithelial cells (IEC) to IFN-γ and TNF-α, two pro-inflammatory mediators associated with gut immunopathology. We found that both BCL-G splice variants — BCL-G_S_ (short) and BCL-G_L_ (long) — were highly expressed in healthy gut tissue, and that their mRNA levels decreased in active inflammatory bowel diseases (for BCL-G_S_) and colorectal cancer (for BCL-G_S/L_). In vitro studies revealed that IFN-γ and TNF-α synergised to upregulate BCL-G_S/L_ and to trigger apoptosis in colonic epithelial cell lines and primary human colonic organoids. Using RNAi, we showed that synergistic induction of IEC death was STAT1-dependent while optimal expression of BCL-G_S/L_ required STAT1, NF-κB/p65 and SWI/SNF-associated chromatin remodellers BRM and BRG1. To test the direct contribution of BCL-G to the effects of IFN-γ and TNF-α on epithelial cells, we used RNAi- and CRISPR/Cas9-based perturbations in parallel with isoform-specific overexpression of BCL-G, and found that BCL-G was dispensable for Th1 cytokine-induced apoptosis of human IEC. Instead, we discovered that depletion of BCL-G differentially affected secretion of inflammatory chemokines CCL5 and CCL20, thus uncovering a non-apoptotic immunoregulatory function of this BCL-2 family member. Taken together, our data indicate that BCL-G may be involved in shaping immune responses in the human gut in health and disease states through regulation of chemokine secretion rather than intestinal apoptosis.

## Introduction

Proteins of the BCL-2 family are central regulators of the intrinsic (mitochondrial) pathway of apoptosis, which is activated by diverse triggers, including death receptor stimulation, DNA damage, growth factor withdrawal or anoikis^1^. BCL-2 family members are characterised by the presence of up to four evolutionarily conserved BCL-2 homology (BH) domains, and are divided into three functional subgroups: the pro-survival guardian proteins (including BCL-2 itself and BCL-X_L_), the BH3-only pro-apoptotic initiator proteins (such as BIM, BID or PUMA) and the multi-domain pro-apoptotic effector proteins (such as BAK)^1^. Together, they regulate the integrity of the mitochondrial outer membrane, whose permeabilisation leads to the release of various apoptogenic factors such as cytochrome c, SMAC or oxidoreductase AIF. These in turn amplify apoptotic signalling through activation of caspase-9, neutralisation of pro-survival IAPs or caspase-independent DNA fragmentation^2^. Although many proteins of the BCL-2 family have a well-understood function in orchestrating apoptosis, there are some ‘orphan’ members for which a defined role in cell death signalling remains unclear^3^.

BCL-G, also known as BCL2L14, is an evolutionarily conserved BCL-2 family member that was originally identified as pro-apoptotic^4^, although this classification was recently challenged^5,6^. The human *BCL-G* gene is located in chromosome 12p12 tumour suppressor locus^7^, and through alternative splicing produces two distinct isoforms: BCL-G_S_ (short) and BCL-G_L_ (long). The short isoform contains only a BH3 domain and when overexpressed is a potent inducer of apoptosis, acting reportedly through sequestration of the pro-survival function of BCL-X_L_^4^. Conversely, BCL-G_L_ possesses both BH2 and BH3 domains, has a limited killing capacity^4^ and thus closely resembles another weakly apoptogenic family member, Bfk^8^. Initial profiling of adult human tissues revealed that expression of BCL-G_S_ was restricted to male reproductive organs, while BCL-G_L_ was detected in various anatomical locations^4^. Little is known, however, about the physiological regulation of BCL-G expression and its functional consequences. The promoter region of *BCL-G* harbours p53-, IRF-1- and STAT1-binding sites, and accordingly BCL-G induction was observed during p53-mediated apoptosis^9^ and following stimulation with type I and type II interferons^10^. Of note, loss of BCL-G attenuated UV-induced apoptosis of breast^11^ and prostate^12^ cancer cells as well as conferred resistance to hypoxia and cisplatin-induced toxicity in kidney epithelial cells^13^, supporting its proposed role in cell death signalling.

However, recent phenotypic analyses of Bcl-G-deficient mice challenged this notion and provided important insight into possible physiological functions of this ‘orphan’ BCL-2 family member^5,6,14^. In mice, the *Bcl-g* gene encodes a single transcript homologous to human BCL-G_L_ and while its tissue distribution pattern closely resembled that of BCL-G_L_, Bcl-g was also highly expressed across the murine gut^5^ including LGR5^+^ colonic stem cells^6^. Bcl-G knockout mice developed normally with intact gastrointestinal homoeostasis and presented no signs of spontaneous (colonic) hyperplasia^5,6^, a functional manifestation often linked to a loss of a pro-apoptotic effector^15^. In particular, splenic dendritic cells lacking Bcl-G remained sensitive to spontaneous ex vivo apoptosis^5^, while data from colitis-associated or genetic models of colorectal cancer showed unperturbed capsase-3 activation in Bcl-G^−/−^ tumours^6^. Taken together, these elegant studies demonstrated that mouse Bcl-G is not a pro-apoptotic regulator.

Multiple signalling pathways control the balance between cellular proliferation, differentiation and cell death, and therefore are critical for maintaining tissue (and ultimately organismal) homoeostasis^16^. However, disruption of this dynamic equilibrium by an abnormal increase in cell death is a pathophysiological hallmark of numerous chronic disease states, including inflammatory bowel diseases (IBD) — ulcerative colitis (UC) and Crohn’s disease (CD) — which are remitting and relapsing multi-factorial inflammatory diseases of the gut^16,17^. An aberrantly high rate of intestinal epithelial cell (IEC) apoptosis in IBD leads to a positive feedback loop of epithelial barrier disruption, microbiota-driven activation of inflammatory responses and further progressive tissue damage, in addition to pathological immune activation through the release of alarmins from dying IEC^18^. This epithelial damage response is often initiated and driven by cytokines associated with Th1 type immunity, in particular by IFN-γ and TNF-α, which are known to induce death of IEC^17^.

In this study, we analysed the expression of BCL-G in human gastrointestinal tissues in health and disease states, and determined its contribution to Th1 cytokine-induced colonic epithelial tissue damage. We report that IFN-γ and TNF-α synergised to induce BCL-G expression and apoptosis in both colonic epithelial cell lines and primary human colonic organoids. Although upregulated during this damage response, human BCL-G — similar to its mouse homologue — was dispensable for cell death. Instead, we discovered a non-apoptotic, immunomodulatory role of BCL-G in regulation of chemokine secretion. When combined with the observed high colonic expression of human BCL-G and its downregulation in gastrointestinal disease, our data suggest that this ‘orphan’ BCL-2 family member may be involved in shaping immune responses in the human gut.

## Materials and methods

### Cell culture

All cell lines were obtained from the American Type Culture Collection and were grown in recommended media supplemented with 10% FBS, 100 IU/ml penicillin and 0.1 mg/ml streptomycin. HT-29 cells (cat# HTB-38) were cultured in McCoy’s 5A medium, DLD-1 cells (cat# CCL-221) in RPMI 1640 medium, LoVo cells (cat# CCL-229) in Ham’s F-12K medium, and L-WRN cells (cat# CRL-3276) in DMEM medium additionally supplemented with 0.5 mg/mL G-418 and 0.5 mg/mL hygromycin B. Cell lines were maintained in humidified 5% CO_2_ atmosphere at 37 °C and routinely tested for mycoplasma with MycoAlert Mycoplasma Detection Kit (cat# LT07-118, Lonza). All cytokine treatments in cell lines were performed in penicillin and streptomycin-supplemented media containing 0.5% FBS.

### Reagents and antibodies

Recombinant human IFN-γ (cat# 300-02) and recombinant human TNF-α (cat# 300-01A) were purchased from Peprotech. For western blotting, the following antibodies were used: anti-β-actin (0.2 μg/ml, cat# A1978, Sigma-Aldrich), anti-BCL-G (0.4 μg/ml, cat# HPA040665, Sigma-Aldrich), anti-caspase-8 (1:1000, cat# 9746, Cell Signaling), anti-caspase-9 (1:1000, cat# 9508, Cell Signaling), anti-cleaved caspase-9 (1:1000, cat# 7237, Cell Signaling), anti-caspase-3 (1:1000, cat# 9662, Cell Signaling), anti-cleaved caspase-3 (1:1000, cat# 9661, Cell Signaling), anti-STAT1 (1:1000, cat# 9172, Cell Signaling), anti-p65 (1:500, cat# sc8008, Santa Cruz Biotechnology), anti-turboGFP (1:2000, cat# TA150041, Origene), goat anti-rabbit immunoglobulins-HRP (cat# P0448, Dako) and rabbit anti-mouse immunoglobulins-HRP (cat# P0260, Dako). The following tGFP-tagged plasmids were purchased from AMS Biotechnology: pCMV6-AC-GFP empty vector (p.Empty, cat# PS100010), BCL-G_L_ expression plasmid (p.BCL-G_L_, cat# RG206404) and BCL-G_S_ expression plasmid (p.BCL-G_S_, cat# RG215114). All other reagents unless specified otherwise were from Sigma-Aldrich.

### BCL-G overexpression

Endotoxin-free plasmid DNA was prepared using PureYield Plasmid Midiprep System (cat# A2492, Promega). DLD-1 cells were transfected with plasmid DNA for 24 or 48 h by forward transfection using Lipofectamine 2000 transfection reagent (cat# 11668027, Invitrogen). For 6-well plates, 2.25 × 10^5^ cells were seeded overnight in 2.5 ml of antibiotic-free media containing 10% FBS. Plasmid DNA-Lipofectamine 2000 transfection complex was prepared the following day by mixing 0.25 ml of 10 ng/μl plasmid DNA solution in Opti-MEM with 0.25 ml of Lipofectamine 2000 diluted 50-fold in Opti-MEM. Resulting 0.5 ml of transfection complex was incubated for 20 min at room temperature and added to seeded cells. After 24 or 48 h, transfected cells were manipulated as indicated in the figures. HT-29 cells were transfected with plasmid DNA using Neon Transfection System (cat# MPK10025, Invitrogen). On the day of transfection, cells were harvested by trypsinisation, washed with PBS and resuspended in Neon resuspension buffer R at the concentration of 1.0 × 10^7^ cell/ml. Per each electroporation, 0.1 ml of the above cell suspension was gently mixed with 5 μg of plasmid DNA and electroporated at 1600 V, 10 ms, 3 pulses. Electroporated cells were immediately transferred to 6-well plates containing 3 ml of pre-warmed antibiotic-free culture medium supplemented with 10% FBS. After 24 h, cells were harvested by trypsinisation, counted and re-seeded in 96-well plates (for cell death and chemokine measurements) or 6-well plates (for western blot analysis). Following overnight adherence, re-seeded cells were treated as indicated in the figures.

### RNAi-mediated gene knockdown

HT-29 cells were transfected with siRNA for 48 h by reverse transfection using Lipofectamine RNAiMAX transfection reagent (cat# 13778150, Invitrogen). The following siRNAs were obtained from Dharmacon as ON-TARGET*plus* SMARTpool reagents: ON-TARGET*plus* Non-targeting Pool (cat# D-001810-10-20), siBCL-G (cat# L-004385-00-0005), siSTAT1 (cat# L-003543-00-0005), sip65 (cat# L-003533-00-0005), siBRM (cat# L-017253-00-0005) and siBRG1 (cat# L-010431-00-0005). Briefly, on the day of transfection cells were harvested by trypsinisation and resuspended in antibiotic-free media containing 10% FBS. For 6-well plates, siRNA-Lipofectamine RNAiMAX transfection complex was prepared by mixing 0.25 ml of 0.25 μM siRNA solution in Opti-MEM with 0.25 ml of Lipofectamine RNAiMAX diluted 33.3-fold in Opti-MEM. Resulting 0.5 ml of transfection complex was incubated for 20 min at room temperature and added to 2 ml of cell suspension (1.5 × 10^5^ cell/ml). After 48 h, transfected cells were treated as indicated in the figures. Knock-downs were validated by either western blotting (for BCL-G, STAT1 and p65) or RT-qPCR (for BCL-G, BRM and BRG1).

### Generation of BCL-G knockout line

HT-29 cells were transfected with gRNA-Cas9 RNP complex for 24 h by reverse transfection using Lipofectamine CRISPRMAX transfection reagent (cat# CMAX00001, Invitrogen). Alt-R CRISPR-Cas9 crRNA targeting exon 2 of BCL-G (TTCGCAGAGCACGCCTGCCA) and Alt-R CRISPR-Cas9 tracrRNA labelled with ATTO 550 (cat# 1075928) were purchased from IDT. Briefly, 1 μM gRNA duplex was prepared by equimolar mixing of crRNA and tracrRNA in Duplex Buffer (cat# 11-01-03-01, IDT), followed by 5 min incubation at 95 °C. For 12-well plates, gRNA-Cas9 RNP complex was prepared by mixing 12 μl of gRNA duplex with 12 μl of Alt-R Cas9 nuclease (cat# 1081058, IDT, 1 μM in Opti-MEM) and 4.8 μl of Cas9 PLUS reagent in Opti-MEM to a final volume of 0.2 ml. After 5 min at room temperature, resulting RNP complex was mixed with 9.6 μl of CRISPRMAX reagent in Opti-MEM to a final volume of 0.4 ml. After 20 min at room temperature, resulting transfection complex was added to 0.8 ml of HT-29 cells resuspended in 10% FBS, antibiotic-free medium at density of 5 × 10^5^ cell/ml. Cells were transfected for 24 h, single cell sorted using FACSAria Fusion (BD Biosciences) and expanded clones were screened for BCL-G knockout by western blotting.

### Human studies

Colonic pinch biopsies were collected with informed consent from adult non-IBD individuals or from patients with Crohn’s disease or ulcerative colitis at Cork University Hospital or Mercy University Hospital. Ethical approval was obtained from the Clinical Research Ethics Committee of the Cork Teaching Hospitals (CREC). Informed consent was obtained from all patients in agreement with the Declaration of Helsinki. All patients with IBD had a confirmed diagnosis based on previous histopathological findings. Active and inactive disease was differentiated by the attending physician on the basis of endoscopic and histological criteria. Healthy controls were comprised of volunteers undergoing investigation of symptoms such as weight loss, anaemia or altered bowel habit.

### Primary human colonic organoid culture

Protocols for crypt isolation and organoid culture were adapted from previously described methods^19,20^. Colonic biopsies from non-IBD individuals were obtained from adult patients at Cork University Hospital and Mercy University Hospital. Before processing, biopsies were stored at 4 °C in Collection Medium (advanced DMEM/F12 (cat# 12634010, Gibco) supplemented with 10% FBS, 100 U/ml penicillin, 0.1 mg/ml streptomycin, 10 mM HEPES (cat# 15630080, Gibco), 2 mM Glutamax (cat# 35050061, Gibco), 1 × Amphotericin B (cat# 11526481, HyClone) and 100 μg/mL gentamicin (cat# G1397, Sigma-Aldrich)). For processing, biopsies were washed in cold PBS supplemented with 1 × Amphotericin B and 100 μg/ml gentamicin. Washed biopsies were dissociated with Gentle Cell Dissociation Reagent (cat# 07174, Stem Cell Technologies) containing 1 × Amphotericin B and 200 μg/mL Gentamicin for 20 min at room temperature with shaking at 180 rpm. Reagent activity was quenched with cold Wash Medium (Advanced DMEM/F12 supplemented with 10% FBS, 100 U/ml penicillin, 0.1 mg/ml streptomycin, 10 mM HEPES and 2 mM Glutamax), and the resulting suspension was passed through a 70 μm filter. The filtered solution was centrifuged for 5 min at 200 rcf and supernatant was discarded. The pellet was resuspended in Wash Medium, transferred to a 1.5 mL tube and centrifuged for 3 min at 400 rcf. Supernatant was removed and the pellet containing isolated crypts was resuspended in Reduced Growth Factor Type 2 BME (cat# 3533-010-02, AMS Biotechnology). For 48-well plates, 20 μl domes of crypt/BME solution were seeded per well and plates were transferred into a cell culture incubator for 20 min. Once polymerised, the domes were overlaid with 300 μl of Proliferation Medium. Proliferation Medium was prepared as follows: Serum Free Medium (Advanced DMEM/F12 supplemented with 10 mM HEPES, 2 mM Glutamax, 1 × N2 supplement (cat# 17502-048, Invitrogen), 1 × B27 supplement (cat# 17504-044, Invitrogen), 1 mM N-acetylcysteine (cat# A9165, Sigma-Aldrich) and 50 ng/ml human recombinant EGF (cat# AF-100-15, PeproTech)) was mixed 1:1 with L-WRN conditioned medium^21^. This 50% L-WRN medium was supplemented with 10 mM nicotinamide (cat# N0636, Sigma-Aldrich), 10 μM Y-27632 (cat# S1049, Selleckchem), 10 μM SB-202190 (cat# S7067, Sigma-Aldrich), 5 μM CHIR-99021 (cat# SML1046, Sigma-Aldrich) and 500 nM A-83-01 (cat# SML0788, Sigma-Aldrich), and filtered using the Steriflip vacuum system (cat# SE1M179M6, Merck). Proliferation Medium was replenished twice a week. For passaging, organoids were removed from BME and dissociated with TrypLE Express (cat# 12604-013, Gibco) supplemented with 10 μM Y-27632, which was then quenched with Wash Medium. The cell solution was centrifuged for 3 min at 400 rcf and supernatant removed. The pellet was resuspended in fresh BME and seeded. Passaging was performed every 1–2 weeks, using a 1:3 split ratio.

### Differentiation of primary human colonic organoids

Organoids were seeded in 48-well plates and overlaid with Proliferation Medium as described above. After 24 h recovery, the differentiated organoid group was switched to Differentiation Medium, the non-differentiated group was kept in Proliferation Medium. Differentiation Medium is Proliferation Medium without nicotinamide, SB-202190 or CHIR-99021 and has reduced L-WRN conditioned media content (5%). Media for both groups were replaced every day for a period of 3 days, after which the organoids were stimulated as indicated in the figures.

### Live microscopy of primary human colonic organoids

Organoids were seeded in 96-well plates using 10 µl BME domes, left to recover for 3 days and stimulated as indicated in the figures. Live transmission light microscopy images were acquired using the EVOS XL Core Cell Imaging System (Invitrogen). Image processing was performed using ImageJ-win64 software. Briefly, images were cropped around the region of interest, processed using the Enhance Local Contrast (CLAHE) plugin, image brightness/contrast was adjusted and a 50 μm scale bar was inserted.

### Crystal violet staining

Cells were seeded in 96-well transparent plates and manipulated as indicated in the figures. At the end-point, cells were washed twice with PBS and fixed with ice-cold methanol for 10 min at −20 °C. After removing the fixative, plates were equilibrated to room temperature and fixed cells were incubated in crystal violet staining solution (0.5 g crystal violet powder, 25 ml methanol and 75 ml distilled water) for 10 min at room temperature. Plates were then washed with distilled water, air-dried overnight and scanned using Odyssey 9120 imaging system (LI-COR). Plates corresponding to consecutive time points within a complete experiment were scanned at the same time. Composite images were generated using ImageJ-win64 software, with splicing indicated by a grey dotted line.

### Measurement of cell viability and caspase-3 activity

Cell viability and caspase-3/7 activity were measured using CellTiter-Glo assay (cat# G7573, Promega) and Caspase-Glo 3/7 assay (cat# G8091, Promega), respectively. For both luminescent assays, organoids or typically 3 × 10^4^ cells were seeded in 96-well plates and treated as indicated in a total volume of 100 μl. At the end-point, 50 μl (or 100 μl in case of organoids) of a corresponding assay reagent were added to plates. Following recommended incubation, 75 μl (or 150 μl in case of organoids) of resulting solution were transferred to 96-well white opaque plates and analysed using Synergy 2 microplate reader (BioTek). Cell death and caspase-3 activation were also monitored by flow cytometry. In this case, 2 × 10^5^ cells were seeded in 24-well plates and treated as indicated for up to 48 h. At each time point, cells (including detached ones) were collected by gentle trypsinisation, washed thrice with PBS and stained with Fixable Viability Stain 660 (cat# 564405, BD Biosciences, 1:2000 in PBS) for 15 min at room temperature. Staining was then quenched by washing cells twice with FACS buffer (2% FBS in PBS), after which cells were incubated in Fixation-Permeabilization solution (cat# 554722, BD Biosciences) for 20 min at 4 °C. Cells were next washed twice with BD Perm/Wash buffer (cat# 554723, BD Biosciences) and stained with anti-active caspase-3 antibody (cat# 561011, BD Biosciences, 1:5 in BD Perm/Wash) for 30 min at 4 °C. After washing twice in BD Perm/Wash, cells were resuspended in FACS-A buffer (2% FBS, 2 mM EDTA in PBS) and analysed using FACSCelesta (BD Biosciences) and FlowJo v10 software.

### Western blotting

Cells were manipulated as indicated in the figures, washed with ice-cold PBS and lysed for 20 min on ice with cold caspase lysis buffer (10 mM Tris–HCl, pH 7.4, 10 mM NaCl, 3 mM MgCl_2_ and 1% NP-40) supplemented with 1 × Halt Protease and Phosphatase Inhibitor Cocktail (cat# 78440, Thermo Scientific) and 0.25 mM AEBSF. Cell lysate was cleared by centrifugation for 20 min at 15,000 rpm and 4 °C, and protein concentration was measured using Pierce BCA Protein Assay Kit (cat# 23225, Thermo Scientific). Typically, 40–50 μg of protein were denatured in 1 × Bolt LDS Sample Buffer (cat# B0007, Invitrogen) supplemented with 1 × Bolt Sample Reducing Agent (cat# B0009, Invitrogen) by heating for 10 min at 70 °C. Samples were separated on Bolt 4–12% Bis-Tris Plus Gels (cat# NW04125BOX, Invitrogen) and transferred to a PVDF membrane (cat# IPVH00010, Millipore) using Criterion blotting system (cat# 1704070, Bio-Rad). Membranes were next blocked with 5% skim milk in TBS-T (0.1% Tween-20 in TBS) for 1.5 h at room temperature and incubated with indicated primary antibodies overnight at 4 °C. For final detection, membranes were probed with a secondary antibody for 1 h at room temperature, followed by detection using WesternBright Quantum HRP substrate (cat# K-12042-D10, Advansta). Western blotting images were acquired using LAS-3000 Imager (Fujifilm) and processed with ImageJ-win64 software (without gel splicing). When required, brightness/contrast was adjusted always for an entire digital image.

### RNA isolation and gene expression analysis

For cell lines, total RNA was isolated using RNeasy Mini Kit (cat# 74106, Qiagen). For organoids, cultures were dissociated with 0.25 ml of Cultrex Organoid Harvesting Solution (cat# 3700-100-01, Bio-Techne) per dome for 1 h at 4 °C with shaking at 180 rpm. Three domes were pooled per condition, collected by centrifugation for 30 s at 10,000 rpm and 4 °C, washed once with cold PBS and lysed in 0.35 ml of RLT buffer containing 1% β-mercaptoethanol. Total RNA was extracted from organoid lysates using RNeasy Micro Kit (cat# 74004, Qiagen) following the recommended on-column DNase digestion protocol. For biopsy samples, colonic pinch biopsies were obtained from adult non-IBD individuals or patients with Crohn’s disease or ulcerative colitis at Cork University Hospital and Mercy University Hospital. Fresh tissue was immediately collected and stored overnight at 4 °C in RNAlater (cat# R0901, Sigma-Aldrich), and subsequently frozen at −80 °C. Frozen tissue was transferred to MagNA Lyser tubes (cat# 03358941001, Roche) containing 0.5 ml of RLT buffer supplemented with 1% β-mercaptoethanol, and homogenised with two cycles of 15 s and 6500 speed using MagNA Lyser instrument (Roche). Total RNA was extracted from biopsy homogenates using RNeasy Mini Kit (cat# 74106, Qiagen), followed by DNase digestion using RNA Clean & Concentrator-5 kit (cat# R1013, Zymo Research). To study tissue-specific gene expression, FirstChoice Human Total RNA Survey Panel (cat# AM6000, Ambion) and additional Human Total RNA samples were used. For cDNA synthesis, 500 ng of total RNA from cell lines (100 ng from organoids, or 200 ng from biopsies) were reverse transcribed using Transcriptor Reverse Transcriptase (cat# 3531287001, Roche) and random hexamer primers. To study cancer-associated gene expression, Colon Cancer TissueScan cDNA array III (cat# HCRT303, OriGene) was used. For gene expression analysis, RT-qPCR assays were designed using the Universal ProbeLibrary System assay design platform (Roche). Primers were purchased from Eurofins Genomics and are listed in Table 1. In addition, the following RealTime ready assays were purchased from Roche: GAPDH (cat# 143641), HPRT1 (cat# 102079), RPL13A (cat# 102119) and TBP (cat# 101145). RT-qPCR reactions containing 1 × Sensi Mix II Probe Kit reaction mix (cat# BIO-83005, Bioline), 500 nM primers, 250 nM Universal ProbeLibrary probe (Roche) and 2.5–10 ng of cDNA were analysed using LightCycler 480 instrument (Roche). Relative gene expression was calculated using the 2^−ΔΔCT^ method^22^, and normalised to β-actin or other stably expressed genes identified by the geNorm algorithm^23^ (HPRT1 and RPL13A for undifferentiated organoids, and TBP and GAPDH for comparing undifferentiated and differentiated organoids).

**Table 1:** Universal ProbeLibrary RT-qPCR assays used in this study.

Gene name	Accession number	Forward primer sequence	Reverse primer sequence	Probe
*BCL-G*_*S*_	NM_030766	CAGGAGATCAGTTGGAAAGAAAG	CTGGTGTCAACCAAGGGAAT	8
*BCL-G*_*L*_	NM_138722	CCCCAGAGAATTCCTTGAGTC	TCATCATCATCTAGGGGGATTT	85
*BCL-G*_*L*_	NM_138722	GCTTTAAGGCTGCCCTTGTA	TCATCGGGTGGTTGTCAAT	100
*STAT1*	NM_007315	GGATCAGCTGCAGAACTGGT	CTGTTCCAATTCCTCCAACTTT	74
*p65/RelA*	NM_021975	ACCGCTGCATCCACAGTT	GATGCGCTGACTGATAGCC	47
*BRM*	NM_003070	GATTCAGCCAGCACACTCCT	GGCGTGGACATCTACCTCTC	53
*BRG1*	NM_001128849	TGGACCAGCACTCCCAAG	CTGGCTGGAACTGGACTAGAG	21
*LGR5*	NM_003667	ACCAGACTATGCCTTTGGAAAC	TTCCCAGGGAGTGGATTCTAT	78
*MUC2*	NM_002457	GCCAGCTCATCAAGGACAG	GCAGGCATCGTAGTAGTGCTG	61
*VIL1*	NM_007127	GCAGCATTACCTGCTCTACGTT	GCTTGATAAGCTGATGCTGTAATTT	71
*ACTB*	NM_001101	ATTGGCAATGAGCGGTTC	CGTGGATGCCACAGGACT	11

For BCL-G overexpression studies, relative mRNA levels of BCL-G_L_ were measured using Universal ProbeLibrary RT-qPCR assay, probe 100.

### Chemokine analysis

At the end-point, cell culture supernatants were cleared by centrifugation for 4 min at 1000 rpm, and stored at −20 °C. Secreted chemokines were measured according to manufacturer’s instructions using the following DuoSet ELISA assays (Bio-Techne): CCL5 (cat# DY278), CCL20 (cat# DY360), CXCL9 (cat# DY392), CXCL10 (cat# DY266) and CXCL11 (cat# DY672).

### Statistical analysis

Experimental results were tested for normality using SPSS Statistics v22 package and analysed for statistical significance using GraphPad Prism v6 software as described in detail in the figure legends. *P* < 0.05, *P* < 0.01 and *P* < 0.001 were considered as statistically significant.

## Results

### BCL-G is differentially expressed in human gastrointestinal tissues in health and disease

**Fig. 1:**
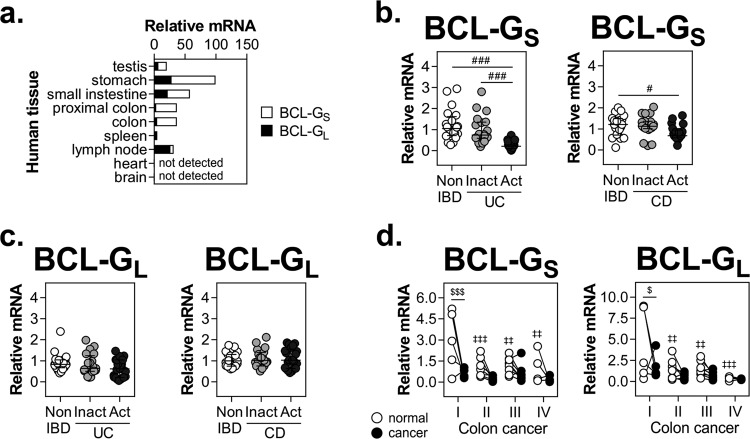
BCL-G is differentially expressed in human gastrointestinal disease. **a** Relative mRNA expression of BCL-G_S/L_ measured by RT-qPCR in the indicated adult human tissues. Relative mRNA expression of **b** BCL-G_S_ and **c** BCL-G_L_ measured by RT-qPCR in colonic biopsy tissues isolated from non-IBD individuals (*n* = 20), patients with inactive (*n* = 20) or active (*n* = 24) ulcerative colitis, and patients with inactive (*n* = 19) or active (*n* = 21) Crohn’s disease. BCL-G_S/L_ expression data in active ulcerative colitis were used for correlation analysis in Fig. 4d. For panels (**b**) and (**c**), data shown include the median with interquartile range. **d** Relative mRNA expression of BCL-G_S/L_ measured by RT-qPCR in TissueScan cDNA array of 24 matched samples (normal, uninvolved colon vs. colorectal cancer) covering clinical stage I (*n* = 5), II (*n* = 7), III (*n* = 8) and IV (*n* = 4). ^#^*p* < 0.05, ^###^*p* < 0.001 (Kruskal–Wallis test followed by Dunn’s multiple comparisons test as indicated), ^$^*p* < 0.05, ^$$$^*p* < 0.001 (repeated measures two-way ANOVA followed by Fisher’s LSD test as indicated), ^‡‡^*p* < 0.01, ^‡‡‡^*p* < 0.001 (repeated measures two-way ANOVA followed by Fisher’s LSD test vs. normal colon, stage I). IBD — inflammatory bowel disease, UC — ulcerative colitis, CD — Crohn’s disease.

Original profiling of adult human tissues suggested that BCL-G_S_ expression was restricted to male reproductive organs, while BCL-G_L_ was detected in a wide range of tissues^4^. Here, we found that apart from testes, both BCL-G transcripts showed prominent expression across the human gastrointestinal tract (stomach, small intestine, proximal colon and colon) (Fig. 1a). High mRNA levels of BCL-G_L_ were also detected in the spleen and lymph nodes, while less immunologically active sites like heart and brain showed no detectable expression of either BCL-G variant. Elevated tissue levels of IFN-γ and TNF-α, and activation of signalling cascades downstream of their respective receptors exacerbate inflammation and tissue damage in IBD^17^. Considering this and high gastrointestinal levels of BCL-G, we profiled its expression in inflamed colonic tissue from patients with UC or CD, two major types of IBD. Compared with non-IBD individuals, BCL-G_S_ was strongly downregulated in colonic mucosal biopsies from patients with active IBD, with an additional decrease in expression between inactive and active disease states in case of UC (Fig. 1b). In contrast, BCL-G_L_ levels remained unchanged in both patient cohorts (Fig. 1c). Given a putative tumour suppressor function of BCL-G^7^, we also examined its expression in human colorectal cancer (CRC). In stage I CRC, levels of both BCL-G transcripts decreased in cancerous tissue compared with patient-matched uninvolved epithelium, and this low tumoural BCL-G_S/L_ expression remained unchanged through clinical grades II–IV (Fig. 1d). While there was no obvious difference in BCL-G_S/L_ expression between uninvolved and cancerous colonic tissue for stages II–IV, we found that BCL-G_S/L_ levels decreased from stage I to stages II–IV in uninvolved epithelium. Collectively, our data highlight transcript-specific differences in BCL-G expression in human gastrointestinal disease, with BCL-G_S_ repression detected in active IBD and downregulation of both BCL-G transcripts observed in colorectal cancer.

### Th1 cytokines IFN-γ and TNF-α synergise to induce BCL-G expression and apoptosis in IEC

**Fig. 2:**
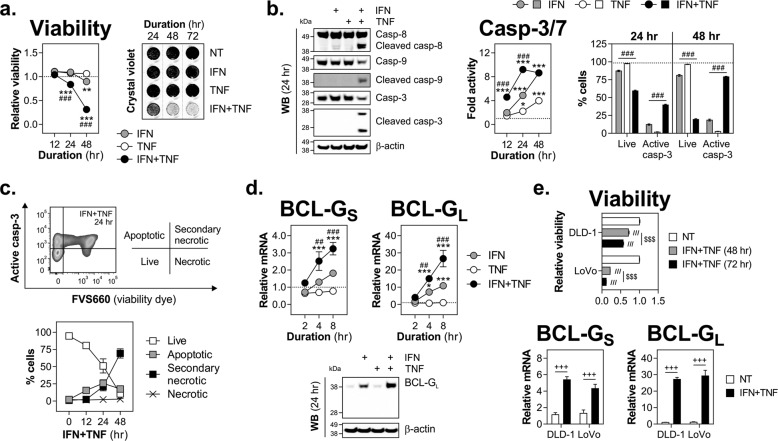
Th1 cytokines IFN-γ and TNF-α synergise to induce BCL-G expression and apoptosis of human IEC. **a**–**d** HT-29 cells were treated with IFN-γ (10 ng/ml), TNF-α (10 ng/ml) or IFN-γ+TNF-α (10 ng/ml each) for the indicated time points. **a** Relative cell viability and crystal violet staining of HT-29 cells treated as indicated. For viability, a black dotted line indicates viability of non-treated cells. For crystal violet staining, grey dotted lines indicate splicing of images. **b** Western blots showing levels of caspase-8, caspase-9 and caspase-3 (total and cleaved) in HT-29 cells treated for 24 h as indicated (left). Caspase-3/7 activity in cytokine-treated HT-29 cells measured over time. A black dotted line indicates caspase-3/7 activity of non-treated cells (middle). Percentage of live cells and active caspase-3^+^ cells following cytokine treatment over time. A black dotted line indicates % of live cells in the non-treated group. Data shown are the mean ± S.D. of *n* = 3 independent experiments in duplicates (right). **c** Cell death profile of HT-29 cells treated over time with IFN-γ+TNF-α, and stained for active caspase-3 and fixable viability dye FVS660. Data shown are the mean ± S.D. of *n* = 3 independent experiments in duplicates. **d** Relative mRNA expression of BCL-G_S/L_ in cytokine-treated HT-29 cells measured by RT-qPCR over time. A black dotted line indicates BCL-G_S/L_ expression in non-treated cells (top). Western blot showing levels of BCL-G_L_ in HT-29 cells treated for 24 h as indicated (bottom). **e** DLD-1 and LoVo cells were treated with IFN-γ+TNF-α (10 ng/ml each) for the indicated time points. Relative cell viability was measured over time (top) and relative mRNA expression of BCL-G_S/L_ was measured by RT-qPCR at 8 h (bottom). Unless specified otherwise, data shown are the mean ± S.E.M. of *n* = 3 independent experiments. *p < 0.05, **p < 0.01 and ***p < 0.001 (two-way ANOVA followed by Tukey’s multiple comparisons test vs. non-treated cells), ^##^*p* < 0.01, ^###^*p* < 0.001 (two-way ANOVA followed by Tukey’s multiple comparisons test vs. IFN-γ), ^///^*p* < 0.001 (one-way ANOVA followed by Tukey’s multiple comparisons test vs. non-treated cells), ^$$$^*p* < 0.001 (one-way ANOVA followed by Tukey’s multiple comparisons test as indicated), ^+++^*p* < 0.001 (unpaired, two-tailed Student’s *t*-test as indicated). NT — non-treated.

Given high colonic levels of BCL-G and its altered expression in the inflamed gut, we determined whether human BCL-G contributes to intestinal epithelial apoptosis. Using an in vitro model of Th1 cytokine-dependent colonic tissue damage, we show that IFN-γ and TNF-α synergised to induce death of HT-29 cells as measured by reduced viability and crystal violet staining over time (Fig. 2a). In contrast, IFN-γ alone displayed a limited killing capacity, while cells exposed to TNF-α remained viable over a three-day period (Fig. 2a). The cytotoxic effect involved activation of initiator caspase-8 and -9 in dually (IFN-γ and TNF-α) treated cells, and was paralleled by steady accumulation of net caspase-3/7 activity and increase in the fraction of cells with active caspase-3 (Fig. 2b). Further kinetic analysis of dually treated cells revealed that cell death induced by IFN-γ and TNF-α progressed from primarily apoptotic at 12 h to being dominated by secondary necrotic cells by 48 h (Fig. 2c). In addition to driving apoptosis during intestinal inflammation^17^, IFN-γ and TNF-α are known to synergistically enhance transcription of pro-inflammatory genes across multiple cell types, including IEC^24^. Gene expression profiling of cytokine-treated HT-29 cells (unpublished data) revealed that BCL-G was upregulated by these two cytokines, pointing towards its putative function in the observed cell death phenotype. Indeed, RT-qPCR analysis confirmed that IFN-γ and TNF-α synergised to upregulate both BCL-G splice variants in HT-29 cells (Fig. 2d). This was also evident at the protein level for BCL-G_L_ (Fig. 2d) as well as in two additional colonic cell lines irrespective of their relative sensitivity to the combination treatment (Fig. 2e). In sum, we demonstrate that BCL-G_S/L_ was highly expressed during Th1 cytokine-induced apoptosis of IEC, and both responses were synergistically regulated by IFN-γ and TNF-α.

### IFN-γ and TNF-α synergise to induce cell death and BCL-G expression in primary human colonic organoids

**Fig. 3:**
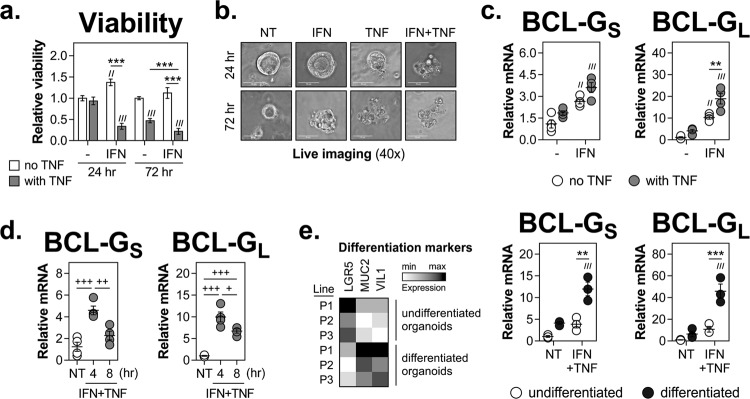
IFN-γ and TNF-α synergise to induce cell death and BCL-G expression in primary human colonic organoids. **a–d** Undifferentiated primary human colonic organoids were treated with IFN-γ (10 ng/ml), TNF-α (10 ng/ml) or IFN-γ+TNF-α (10 ng/ml each) for the indicated time points. **a** Relative organoid viability measured at 24 and 72 h in *n* = 5 organoid lines. **b** Representative live images of one of the organoid lines used in panel (**a**), and treated as indicated. **c** Relative mRNA expression of BCL-G_S/L_ measured by RT-qPCR at 4 h after cytokine treatment in *n* = 4 organoid lines. **d** Relative mRNA expression of BCL-G_S/L_ measured by RT-qPCR at 4 and 8 h after combined cytokine treatment in *n* = 4 organoid lines. **e** Primary human colonic organoids undifferentiated or differentiated for 3 days were treated with IFN-γ+TNF-α (10 ng/ml each) for 4 h. Heatmap showing ranked relative mRNA expression of differentiation markers (LGR5, MUC2 and VIL1) across *n* = 3 organoid lines (left). Relative mRNA expression of BCL-G_S/L_ measured by RT-qPCR in *n* = 3 organoid lines (right). All organoid lines were generated from colonic biopsies from non-IBD individuals. Data shown are the mean ± S.E.M. ^//^*p* < 0.01 and ^///^*p* < 0.001 (two-way ANOVA followed by Tukey’s multiple comparisons test vs. undifferentiated, non-treated organoids), **p < 0.01 and ***p < 0.001 (two-way ANOVA followed by Tukey’s multiple comparisons test as indicated), ^+^*p* < 0.05, ^++^*p* < 0.01 and ^+++^*p* < 0.001 (one-way ANOVA followed by Tukey’s multiple comparisons test as indicated). NT — non-treated, P — patient-derived organoid line.

To validate the above findings in a human primary 3D model of an intestinal damage response, we studied cell death and BCL-G expression in patient-derived colonic organoid cultures, which were established from epithelial crypts isolated from non-IBD colonic mucosal biopsy tissue. Similar to the pattern found in 2D cell line cultures, the combination of IFN-γ and TNF-α synergised to induce death of colonic organoids (Fig. 3a, b). This was initially triggered by the synergistic interaction of IFN-γ and TNF-α, to be primarily driven by TNF-α on day 3 of cytokine treatment. In addition, both BCL-G transcripts were strongly upregulated by IFN-γ and TNF-α in primary colonic organoids (Fig. 3c) albeit at a higher transcriptional rate, with relative expression of BCL-G_S/L_ peaking at 4 h after cytokine treatment (Fig. 3d). We next tested whether the differentiation of organoids impacted the ability of Th1 cytokines to upregulate BCL-G. As shown in Fig. 3e, upon differentiation all three organoid lines expressed higher levels of MUC2 and VIL1 while downregulating LGR5, indicative of the enrichment for differentiated goblet cells and enterocytes at the expense of intestinal stem cells. Those differentiated organoids were more proficient at inducing BCL-G_S/L_ expression following IFN-γ and TNF-α treatment than the undifferentiated cultures (Fig. 3e). In conclusion, both phenotypic responses to Th1 cytokines — synergistic induction of cell death and upregulation of BCL-G_S/L_ — were fully retained in human primary colonic organoids.

### STAT1, NF-κB/p65 and SWI/SNF chromatin remodelling complex are required for Th1 cytokine-induced expression of BCL-G

**Fig. 4:**
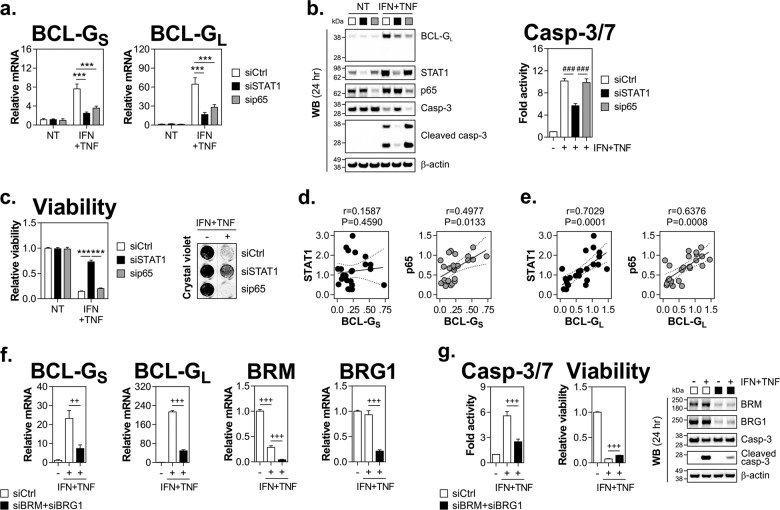
STAT1, NF-κB/p65 and SWI/SNF chromatin remodelling complex are required for BCL-G induction by IFN-γ and TNF-α. **a**–**c** HT-29 cells transfected with a non-targeting siRNA (siCtrl), siRNA targeting STAT1 (siSTAT1) or NF-κB/p65 (sip65) were stimulated 48 h later with IFN-γ (25 ng/ml) and TNF-α (50 ng/ml) for various time points. **a** Relative mRNA expression of BCL-G_S/L_ as measured by RT-qPCR at 4 h. **b** Western blots showing levels of BCL-G_L_, STAT1, NF-κB/p65 and caspase-3 (total and cleaved) 24 h after cytokine treatment (left). Caspase-3/7 activity as measured at 24 h (right). **c** Relative cell viability and crystal violet staining of HT-29 cells treated as indicated for 48 and 72 h, respectively. For crystal violet staining, a grey dotted line indicates splicing of images. **d** Spearman correlation between BCL-G_S_ and STAT1 (left) or NF-κB/p65 (right) relative mRNA levels in colonic biopsy tissues of patients with active ulcerative colitis. **e** Spearman correlation between BCL-G_L_ and STAT1 (left) or NF-κB/p65 (right) relative mRNA levels in colonic biopsy tissues of patients with active ulcerative colitis. **f**, **g** HT-29 cells transfected with a non-targeting siRNA (siCtrl) or siRNAs targeting BRM (siBRM) and BRG1 (siBRG1) were stimulated 48 h later with IFN-γ (25 ng/ml) and TNF-α (50 ng/ml) for various time points. **f** Relative mRNA expression of BCL-G_S/L_, BRM and BRG1 as measured by RT-qPCR at 8 h. **g** Caspase-3/7 activity at 24 h (left), relative viability at 48 h (middle) and western blots showing levels of BRM, BRG1 and caspase-3 cleavage at 24 h (right) in HT-29 cells treated as indicated. Data shown are the mean ± S.E.M. of *n* = 3 independent experiments. ***p < 0.001 (two-way ANOVA followed by Tukey’s multiple comparisons test as indicated), ^###^*p* < 0.001 (one-way ANOVA followed by Tukey’s multiple comparisons test as indicated), ^++^*p* < 0.01, ^+++^*p* < 0.001 (unpaired, two-tailed Student’s *t*-test as indicated). NT — non-treated, *r* — Spearman correlation coefficient, *P* — two-tailed *p*-value of Spearman correlation.

Transcriptional synergism downstream of IFN-γ and TNF-α receptor engagement is known to be mediated by the co-operative action of STAT1 and NF-κB/p65 subunit (also known as RelA), two transcription factors (TFs) activated by IFN-γ and TNF-α, respectively^25^. We found that RNAi knockdown of either TF significantly reduced expression of BCL-G_S/L_ upon cytokine treatment, both at the mRNA and protein levels (Fig. 4a, b). In terms of cell fate decisions, loss of STAT1 had a strong protective effect while depletion of NF-κB/p65 failed to rescue HT-29 cells from apoptosis (Fig. 4b, c). To further support the notion that these TFs contribute to BCL-G expression under inflammatory conditions, we measured their levels in inflamed colonic biopsies from patients with active UC. We found a strong positive correlation between relative mRNA levels of BCL-G_S_ and NF-κB/p65 (Fig. 4d), and BCL-G_L_ and either STAT1 or NF-κB/p65 (Fig. 4e). Together, these data point towards STAT1 and NF-κB/p65 as important regulators of BCL-G_S/L_ expression in inflamed epithelium, with STAT1 alone being also required for propagation of the synergistic cell death signal delivered by IFN-γ and TNF-α.

Inducible expression of inflammatory genes is tightly controlled and often involves stimulus-dependent histone modifications and chromatin remodelling^26^. This was reported for a number of IFN-γ-dependent genes, whose optimal induction required the SWI/SNF chromatin remodelling complex^27–29^. In particular, combined depletion of two catalytic subunits of the SWI/SNF complex — BRM and BRG1 — impaired the inflammatory response to LPS in mouse macrophages^30^. Here, we demonstrate that BRM/BRG1 double knockdown in HT-29 cells decreased expression of both BCL-G variants following IFN-γ and TNF-α treatment (Fig. 4f). The concurrent loss of BRM/BRG1 also attenuated apoptosis as indicated by reduced caspase-3/7 activity and caspase-3 cleavage in dually treated cells (Fig. 4g). Collectively, these data highlight a role for STAT1 and SWI/SNF-mediated chromatin remodelling in positive regulation of inducible BCL-G_S/L_ expression and epithelial cell apoptosis in response to IFN-γ and TNF-α.

### BCL-G regulates secretion of chemokines but not apoptosis driven by IFN-γ and TNF-α in IEC

**Fig. 5:**
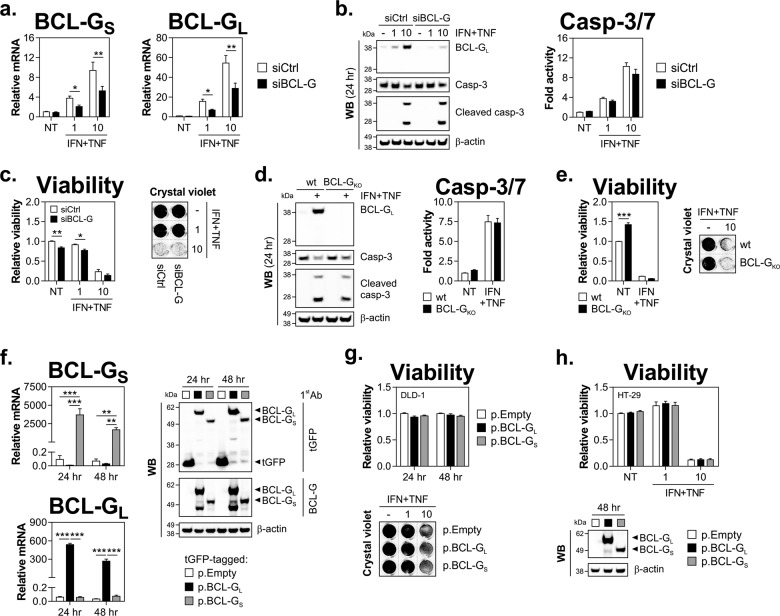
BCL-G is dispensable for apoptosis induced by IFN-γ and TNF-α in human IEC. **a–c** HT-29 cells transfected with a non-targeting siRNA (siCtrl) or siRNA targeting BCL-G (siBCL-G) were stimulated 48 h later with IFN-γ+TNF-α (1 or 10 ng/ml each) for various time points. **a** Relative mRNA expression of BCL-G_S/L_ as measured by RT-qPCR at 8 h after cytokine treatment. **b** Western blots showing levels of BCL-G_L_ and caspase-3 (total and cleaved) at 24 h after cytokine treatment (left). Caspase-3/7 activity as measured at 24 h (right). **c** Relative cell viability and crystal violet staining of HT-29 cells treated as indicated for 48 and 72 h, respectively. **d** Western blots showing levels of BCL-G_L_ and caspase-3 (total and cleaved) in wild-type or BCL-G knockout (BCL-G_KO_) HT-29 cells treated with IFN-γ+TNF-α (10 ng/ml each) for 24 h (left). Caspase-3/7 activity was measured at 24 h (right). **e** Relative cell viability and crystal violet staining of wild-type or BCL-G_KO_ HT-29 cells treated with IFN-γ+TNF-α (10 ng/ml each) for 72 h. **f**, **g** DLD-1 cells were transfected with a tGFP-tagged empty vector (p.Empty) or BCL-G_S_ (p.BCL-G_S_) or BCL-G_L_ (p.BCL-G_L_) expression plasmids for the indicated time points. **f** Relative mRNA expression of BCL-G_S/L_ as measured by RT-qPCR (left) and western blots showing protein levels of BCL-G_S/L_ (right) at 24 and 48 h. **g** Relative viability of DLD-1 cells at 24 and 48 h after transfection (left) and crystal violet staining of DLD-1 cells transfected for 24 h, followed by treatment with IFN-γ+TNF-α (1 or 10 ng/ml each) for 72 h (right). **h** Relative viability of HT-29 cells plasmid transfected for 48 h, followed by treatment with IFN-γ+TNF-α (1 or 10 ng/ml each) for 72 h (top). Western blot showing protein levels of BCL-G_S/L_ at 48 h after plasmid transfection (bottom). Data shown are the mean ± S.E.M. of at least *n* = 3 independent experiments. *p < 0.05, **p < 0.01 and ***p < 0.001 (two-way ANOVA followed by Tukey’s multiple comparisons test as indicated). For crystal violet staining, grey dotted lines indicate splicing of images. NT — non-treated, wt — wild-type.

BCL-G was first identified as a pro-apoptotic member of the BCL-2 protein family^4^. In this study, we found that under inflammatory conditions reduced expression of BCL-G_S/L_ was linked to decreased caspase activation in the context of STAT1 or BRM/BRG1 depletion. Therefore, we investigated whether BCL-G directly regulated IFN-γ and TNF-α-induced death of human IEC. As shown in Fig. 5a, b, targeting BCL-G_S/L_ by RNAi had no inhibitory effect on caspase-3 processing or caspase-3/7 activity in dually treated HT-29 cells. Ultimately, loss of BCL-G failed to protect those cells from IFN-γ and TNF-α-induced apoptosis (Fig. 5c). This observation was recapitulated in BCL-G knockout (BCL-G_KO_) HT-29 cells, which proved as proficient at capase-3/7 activation (Fig. 5d) and as susceptible to Th1 cytokine-driven cell death (Fig. 5e) as the parental line. To complement both loss-of-function approaches, we overexpressed each BCL-G splice variant in DLD-1 and HT-29 cells to asses any potential isoform-specific effects on cell fate decisions. We found that ectopic expression of BCL-G isoforms had no intrinsic killing activity, and also failed to sensitise cells to either a sub-lethal (1 ng/ml) or toxic (10 ng/ml) dose of IFN-γ and TNF-α (Fig. 5f–h). Together, this demonstrated that BCL-G was dispensable for Th1 cytokine-induced apoptosis of human IEC.

**Fig. 6:**
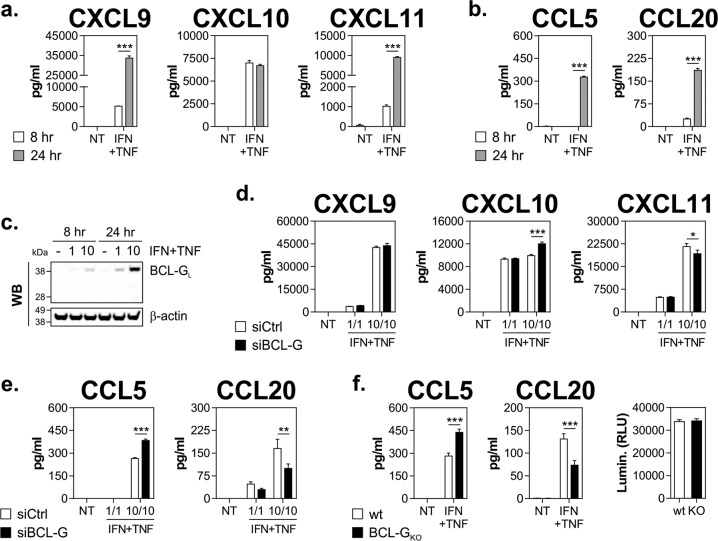
BCL-G regulates induction of pro-inflammatory chemokines by IFN-γ and TNF-α in human IEC. **a**–**c** HT-29 cells were stimulated with IFN-γ+TNF-α (1 or 10 ng/ml each) for 8 and 24 h. **a** Concentration of CXCL9, CXCL10, and CXCL11 measured in cell culture supernatants. **b** Concentration of CCL5 and CCL20 measured in cell culture supernatants. **c** Western blot showing levels of BCL-G_L_ in HT-29 cells treated as indicated. **d**, **e** HT-29 cells transfected with a non-targeting siRNA (siCtrl) or siRNA targeting BCL-G (siBCL-G) were stimulated 48 h later with IFN-γ+TNF-α (1 or 10 ng/ml each) for 24 h. **d** Concentration of CXCL9, CXCL10 and CXCL11 measured in cell culture supernatants following BCL-G knockdown. **e** Concentration of CCL5 and CCL20 measured in cell culture supernatants following BCL-G knockdown. **f** Wild-type or BCL-G knockout (BCL-G_KO_) HT-29 cells were treated with IFN-γ+TNF-α (10 ng/ml each) for 24 h. Concentration of CCL5 and CCL20 measured in cell culture supernatants (left). Luminescent Cell-Titer Glo signal of wild-type and BCL-G_KO_ HT-29 cells after 24 h culture (right). Data shown are the mean ± S.E.M. of at least *n* = 3 independent experiments. *p < 0.05, **p < 0.01 and ***p < 0.001 (two-way ANOVA followed by Tukey’s multiple comparisons test as indicated). NT — non-treated, wt — wild-type.

Based on the interaction between mouse Bcl-G and members of the TRAPP complex, Giam and colleagues postulated that Bcl-G may play a role in vesicle trafficking and protein transport rather than regulation of apoptosis^5^. This hypothesis was recently reinforced by the identification of a functionally similar interactome of BCL-G in human cells^31^. This prompted us to test whether loss of BCL-G could alter induction of inflammatory chemokines by IFN-γ and TNF-α in HT-29 cells. To this end, we measured protein expression of ‘early’ chemokines, which accumulate at high levels prior to BCL-G_L_ protein (CXCL9–11) and ‘late’ chemokines, whose expression follows BCL-G_L_ kinetics (CCL5 and 20) (Fig. 6a–c). As shown in Fig. 6d, secretion of early chemokines was only marginally affected by BCL-G loss. Instead, CCL5 levels increased by nearly 50% following BCL-G knockdown in dually treated cells, while secreted CCL20 displayed an inverse but equally robust behaviour (Fig. 6e). Both these results were fully recapitulated in the BCL-G_KO_ line, which showed no obvious changes in steady-state proliferation compared with the paternal line (Fig. 6f). Consistent with the loss-of-function approach, ectopic expression of BCL-G in cell lines reduced CCL5 levels and increased CCL20 production following the combination treatment (Supplementary Fig. 1). In summary, we found that while it was dispensable for apoptosis, BCL-G differentially regulated secretion of select inflammatory chemokines by human IEC in response to IFN-γ and TNF-α.

## Discussion

Previous reports indicated that immune-related signalling pathways such as those initiated by TLR9 stimulation^5^ and interferons^10^ might be largely responsible for inducible expression of BCL-G. Here, we investigated transcriptional regulation of human BCL-G and its function in dying IEC using an in vitro model of Th1 cytokine-induced tissue damage in the gut. We found that both BCL-G splice variants were synergistically upregulated by the combination of IFN-γ and TNF-α in colonic cell lines and primary human organoids. Multiple mechanisms of transcriptional synergy between IFN-γ and TNF-α have been reported, including enhanced degradation of NF-κB inhibitory proteins^32^ or increased binding of STAT1 and NF-κB/p65 to their cognate regulatory sequences^25^. Using RNAi, we found that both STAT1 and NF-κB/p65 were required for optimal induction of BCL-G_S/L_ by IFN-γ and TNF-α. The fact that TNF-α alone failed to upregulate BCL-G_S/L_ yet loss of NF-κB/p65 reduced BCL-G_S/L_ levels in stimulated cells indicated a potential role for chromatin remodelling. Indeed, BCL-G_S/L_ expression in activated IEC decreased following concurrent loss of BRM and BRG1, two catalytic subunits of the SWI/SNF chromatin remodelling complex known to control IFN-γ-dependent transcriptional programmes^27–29^. It is therefore possible that IFN-γ through STAT1 binding to the *BCL-G* promoter facilitated recruitment of SWI/SNF to remodel the local chromatin environment, thereby making previously unavailable binding sites accessible to NF-κB/p65, which in turn co-operated with STAT1 to drive maximal expression of BCL-G.

BCL-G was originally reported to trigger apoptosis when overexpressed in non-human primate cells^4^, and this response could be partially blocked by MELK-dependent phosphorylation of BCL-G^33^. The killing capacity of endogenous BCL-G was supported by subsequent studies in human osteosarcoma^9^, breast^11^ and prostate^12^ cancer cell lines, which showed a protective effect of BCL-G depletion during p53 activation or UV irradiation. Other reports, while suggesting a pro-apoptotic role of BCL-G, lacked a conclusive support for the observed cell death being directly controlled by BCL-G alone^34^. Here, we report that in human IEC and intestinal organoids expression of BCL-G_S/L_ paralleled induction of apoptosis by IFN-γ and TNF-α, where it converged with activation of caspase-9, suggesting engagement of the mitochondrial pathway. However, targeted depletion of BCL-G had no impact on effector caspase activity, and ultimately failed to protect simulated IEC from apoptosis. In line with this, neither BCL-G variant when overexpressed was cytotoxic, even when coupled with increasing concentrations of IFN-γ and TNF-α. In sum, our data demonstrate that human BCL-G had no intrinsic pro-apoptotic activity in IEC under steady-state conditions, and was dispensable for Th1 cytokine-induced apoptosis. This is functionally consistent with recent observations from Nguyen and colleagues, who showed that loss of mouse Bcl-g had no protective effect on the intestinal epithelium under acute and chronic inflammatory conditions or following irradiation-induced injury^6^.

Instead, we found that either transient or stable loss of BCL-G had a consistent immunomodulatory effect in stimulated epithelia. Specifically, depletion of BCL-G in IEC increased levels of secreted CCL5 by nearly 50%, while having an opposite but equally robust effect on CCL20. This suggested that rather than regulating apoptosis BCL-G may be involved in co-ordination of cellular protein transport. Our hypothesis is supported by the findings from Giam and colleagues^5^, who identified components of the TRAPPII complex (TRAPPC3/4/5) as endogenous interactors of mouse Bcl-G in gastrointestinal epithelial cells, while an analogous interactome was recently described for human BCL-G^31^. The mammalian TRAPPII complex controls vesicle-mediated ER-to-Golgi transport^35^ and thus its putative perturbation following BCL-G loss may have altered chemokine secretion as observed in our study. Of note, TRAPPC4 was shown to regulate activity and nuclear localisation of ERK1/2 in human colorectal cancer cells^36^, while Bcl-G itself is known to restrict LPS-induced ERK1/2 signalling in mouse macrophages^37^. Another example of an immunoregulatory function of BCL-G comes from a 2018 study, which identified BCL-G as a novel restriction factor for HIV infection in humans during the course IFN-α2b therapy^38^. Most recently, Nguyen and colleagues reported that loss of Bcl-G aggravated development of colitis-associated cancer in mice, likely through disruption of tissue-protective functions of IEC as intratumoural infiltration by CD45^+^ leucocytes was comparable between wild-type and Bcl-G^−/−^ hosts^6^. In the same study, they also found that Bcl-g deficiency had no impact on influx of T-cells or macrophages into colonic lamina propria in the acute colitis model. Data presented herein extend our current knowledge of this emerging immunomodulatory activity of BCL-G, by highlighting its role in differential control of chemokine secretion in human IEC, which may represent an evolutionary gain-of-function event. It is interesting to note that an RNAi screen for modulators of NOD1 signalling identified a similar non-apoptotic role for another BCL-2 family member BID in intestinal inflammation, whereby HT-29 cells depleted for BID or macrophages from Bid^−/−^ mice showed blunted cytokine secretion^39^.

Finally, we found that both BCL-G splice variants showed prominent expression in the gastrointestinal tract, which expanded the initial characterisation of BCL-G tissue distribution pattern in humans^4^. While previous studies described BCL-G_L_ downregulation in UC^6^, we found no evidence of altered BCL-G_L_ expression in IBD. This might reflect critical differences in methodologies (microarray^6^ vs. transcript-specific RT-qPCR) or inter-cohort variability. Instead, we report that BCL-G_S_ mRNA levels decreased in mucosal biopsies of patients with active UC and CD. This was at odds with our in vitro data and could be explained by contribution of other mediators in a local tissue environment to BCL-G expression. Alternatively, since both splice variants were also detected in lymph nodes it is possible that the observed changes could reflect transcriptional dynamics and/or fluctuating distribution of infiltrating immune cells at the biopsied site. Indeed, mucosal T-cells from patients with IBD display altered levels of other BCL-2 family members, such as BCL-2 or BAX^40,41^. We also discovered that BCL-G_S/L_ expression was reduced in stage I CRC when compared with patient-matched uninvolved colonic tissue. Intriguingly, while tumoural BCL-G_S/L_ expression remained low across all clinical grades, mRNA levels of BCL-G_S/L_ decreased in uninvolved epithelium from stage I to stages II–IV. This suggests that initial reduction in BCL-G_S/L_ was intrinsic to cancerous tissue, and therefore may represent a genetic lesion indicative of the proposed tumour suppressor function of BCL-G^7^. Instead, progressive changes in a tumour microenvironment are more likely to underpin the observed widespread downregulation of BCL-G_S/L_ in the diseased gut.

In conclusion, although upregulated during the course of IFN-γ and TNF-α-induced apoptosis, BCL-G was dispensable for death of IEC. Instead, we describe a previously unreported non-apoptotic function of BCL-G in regulation of chemokine secretion from IEC in response to Th1 cytokines. Because of this immunomodulatory role in activated epithelia, our data suggest that isoform-specific changes in BCL-G expression in IBD and colorectal cancer may therefore reflect and perhaps contribute to altered immune signatures in these disease states.

## Supplementary information

Suppl Figure 1 legend

Suppl Figure 1

## References

[1] Czabotar PE, Lessene G, Strasser A, Adams JM (2014). Control of apoptosis by the BCL-2 protein family: implications for physiology and therapy. Nat. Rev. Mol. Cell Biol..

[2] van Loo G (2002). The role of mitochondrial factors in apoptosis: a Russian roulette with more than one bullet. Cell Death Differ..

[3] Chipuk JE, Moldoveanu T, Llambi F, Parsons MJ, Green DR (2010). The BCL-2 family reunion. Mol. Cell.

[4] Guo B, Godzik A, Reed JC (2001). Bcl-G, a novel pro-apoptotic member of the Bcl-2 family. J. Biol. Chem..

[5] Giam M, Okamoto T, Mintern JD, Strasser A, Bouillet P (2012). Bcl-2 family member Bcl-G is not a proapoptotic protein. Cell Death Dis..

[6] Nguyen Paul M., Dagley Laura F., Preaudet Adele, Lam Nga, Giam Maybelline, Fung Ka Yee, Aizel Kaheina, van Duijneveldt Gemma, Tan Chin Wee, Hirokawa Yumiko, Yip Hon Yan K., Love Christopher G., Poh Ashleigh R., Cruz Akshay D’, Burstroem Charlotte, Feltham Rebecca, Abdirahman Suad M., Meiselbach Kristy, Low Ronnie Ren Jie, Palmieri Michelle, Ernst Matthias, Webb Andrew I., Burgess Tony, Sieber Oliver M., Bouillet Philippe, Putoczki Tracy L. (2019). Loss of Bcl-G, a Bcl-2 family member, augments the development of inflammation-associated colorectal cancer. Cell Death & Differentiation.

[7] Montpetit A, Boily G, Sinnett D (2002). A detailed transcriptional map of the chromosome 12p12 tumour suppressor locus. Eur. J. Hum. Genet.

[8] Coultas L (2003). Bfk: a novel weakly proapoptotic member of the Bcl-2 protein family with a BH3 and a BH2 region. Cell Death Differ..

[9] Miled C, Pontoglio M, Garbay S, Yaniv M, Weitzman JB (2005). A genomic map of p53 binding sites identifies novel p53 targets involved in an apoptotic network. Cancer Res..

[10] Zhang XN, Liu JX, Hu YW, Chen H, Yuan ZH (2006). Hyper-activated IRF-1 and STAT1 contribute to enhanced interferon stimulated gene (ISG) expression by interferon alpha and gamma co-treatment in human hepatoma cells. Biochim Biophys. Acta.

[11] Pickard MR (2009). Dysregulated expression of Fau and MELK is associated with poor prognosis in breast cancer. Breast Cancer Res..

[12] Pickard MR, Edwards SE, Cooper CS, Williams GT (2010). Apoptosis regulators Fau and Bcl-G are down-regulated in prostate cancer. Prostate.

[13] Zynda ER (2016). An RNA interference screen identifies new avenues for nephroprotection. Cell Death Differ..

[14] Giam M (2012). Detection of Bcl-2 family member Bcl-G in mouse tissues using new monoclonal antibodies. Cell Death Dis..

[15] Adams JM, Cory S (2007). The Bcl-2 apoptotic switch in cancer development and therapy. Oncogene.

[16] Koch S, Nusrat A (2012). The life and death of epithelia during inflammation: lessons learned from the gut. Annu Rev. Pathol..

[17] Neurath MF (2014). Cytokines in inflammatory bowel disease. Nat. Rev. Immunol..

[18] Pasparakis M, Vandenabeele P (2015). Necroptosis and its role in inflammation. Nature.

[19] Sato T (2011). Long-term expansion of epithelial organoids from human colon, adenoma, adenocarcinoma, and Barrett’s epithelium. Gastroenterology.

[20] VanDussen KL (2015). Development of an enhanced human gastrointestinal epithelial culture system to facilitate patient-based assays. Gut.

[21] Miyoshi H, Stappenbeck TS (2013). In vitro expansion and genetic modification of gastrointestinal stem cells in spheroid culture. Nat. Protoc..

[22] Livak KJ, Schmittgen TD (2001). Analysis of relative gene expression data using real-time quantitative PCR and the 2(-Delta Delta C(T)) Method. Methods.

[23] Vandesompele J (2002). Accurate normalization of real-time quantitative RT-PCR data by geometric averaging of multiple internal control genes. Genome Biol..

[24] Dwinell MB, Lugering N, Eckmann L, Kagnoff MF (2001). Regulated production of interferon-inducible T-cell chemoattractants by human intestinal epithelial cells. Gastroenterology.

[25] Ohmori Y, Schreiber RD, Hamilton TA (1997). Synergy between interferon-gamma and tumor necrosis factor-alpha in transcriptional activation is mediated by cooperation between signal transducer and activator of transcription 1 and nuclear factor kappaB. J. Biol. Chem..

[26] Medzhitov R, Horng T (2009). Transcriptional control of the inflammatory response. Nat. Rev. Immunol..

[27] Pattenden SG, Klose R, Karaskov E, Bremner R (2002). Interferon-gamma-induced chromatin remodeling at the CIITA locus is BRG1 dependent. EMBO J..

[28] Ni Z (2005). Apical role for BRG1 in cytokine-induced promoter assembly. Proc. Natl Acad. Sci. USA.

[29] Ni Z, Abou El Hassan M, Xu Z, Yu T, Bremner R (2008). The chromatin-remodeling enzyme BRG1 coordinates CIITA induction through many interdependent distal enhancers. Nat. Immunol..

[30] Ramirez-Carrozzi VR (2006). Selective and antagonistic functions of SWI/SNF and Mi-2beta nucleosome remodeling complexes during an inflammatory response. Genes Dev..

[31] Hubel P (2019). A protein-interaction network of interferon-stimulated genes extends the innate immune system landscape. Nat. Immunol..

[32] Cheshire JL, Baldwin AS (1997). Synergistic activation of NF-kappaB by tumor necrosis factor alpha and gamma interferon via enhanced I kappaB alpha degradation and de novo I kappaBbeta degradation. Mol. Cell Biol..

[33] Lin ML, Park JH, Nishidate T, Nakamura Y, Katagiri T (2007). Involvement of maternal embryonic leucine zipper kinase (MELK) in mammary carcinogenesis through interaction with Bcl-G, a pro-apoptotic member of the Bcl-2 family. Breast Cancer Res..

[34] Watanabe J, Nakagawa M, Watanabe N, Nakamura M (2013). Ubiquitin-like protein MNSFbeta covalently binds to Bcl-G and enhances lipopolysaccharide/interferon gamma-induced apoptosis in macrophages. FEBS J..

[35] Sacher M, Kim YG, Lavie A, Oh BH, Segev N (2008). The TRAPP complex: insights into its architecture and function. Traffic.

[36] Zhao SL (2011). TRAPPC4-ERK2 interaction activates ERK1/2, modulates its nuclear localization and regulates proliferation and apoptosis of colorectal cancer cells. PLoS ONE.

[37] Nakamura M, Yamaguchi S (2006). The ubiquitin-like protein MNSFbeta regulates ERK-MAPK cascade. J. Biol. Chem..

[38] El-Diwany R (2018). CMPK2 and BCL-G are associated with type 1 interferon-induced HIV restriction in humans. Sci. Adv..

[39] Yeretssian G (2011). Non-apoptotic role of BID in inflammation and innate immunity. Nature.

[40] Ina K (1999). Resistance of Crohn’s disease T cells to multiple apoptotic signals is associated with a Bcl-2/Bax mucosal imbalance. J. Immunol..

[41] Itoh J, de La Motte C, Strong SA, Levine AD, Fiocchi C (2001). Decreased Bax expression by mucosal T cells favours resistance to apoptosis in Crohn’s disease. Gut.

