# Supplementary material for: Human BCL-G regulates secretion of inflammatory chemokines but is dispensable for induction of apoptosis by IFN-γ and TNF-α in intestinal epithelial cells
Source: Cell Death Dis. 2020 Jan 27;11(1):68. doi: 10.1038/s41419-020-2263-0 (PMC6985252; doi:10.1038/s41419-020-2263-0)
Supplement: Supplementary file 1 — Suppl Figure 1 legend [file 41419_2020_2263_MOESM1_ESM.docx]

**Supplementary Figure LEGENDS**

**Human BCL-G regulates secretion of inflammatory chemokines but is dispensable for induction of apoptosis by IFN‑γ and TNF‑α in intestinal epithelial cells**

**Running title:** BCL-G regulates secretion of chemokines in IEC

Jerzy A. Woznicki^1^, Peter Flood^1^, Milan Bustamante-Garrido^1^, Panagiota Stamou^1^, Gerry Moloney^1^, Aine Fanning^1^, Syed Akbar Zulquernain^1,2^, Jane McCarthy^3^, Fergus Shanahan^1,2^, Silvia Melgar^1^, and Ken Nally^1,4,5^

^1^ APC Microbiome Ireland, University College Cork, Cork, Ireland

^2^ Department of Medicine, University College Cork, Cork, Ireland

^3^ Department of Gastroenterology, Mercy University Hospital, Cork, Ireland

^4^ School of Biochemistry & Cell Biology, University College Cork, Cork, Ireland

^5^ Corresponding author: [k.nally@ucc.ie](mailto:k.nally@ucc.ie)

**Supplementary Figure 1. Effect of BCL-G over-expression on chemokine induction by IFN-γ and TNF-α in human IEC.** HT-29 and DLD‑1 cells were transfected with a tGFP-tagged empty vector (p.Empty), BCL-G_S_ (p.BCL‑G_S_) or BCL‑G_L_ (p.BCL‑G_L_) expression plasmids for 48 hr (HT-29) or 24 hr (DLD-1), followed by treatment with IFN-γ+TNF-α (1 or 10 ng/ml each) for 24 hr. **(a)** Concentration of CCL5 measured in cell culture supernatants. **(b)** Concentration of CCL20 measured in cell culture supernatants. Data shown are the mean ± S.E.M. of n=3 independent experiments. ^*^p<0.05 and ^**^p<0.01 (two-way ANOVA followed by Tukey's multiple comparisons test as indicated). NT – non-treated
