# Supplementary figures and images for: Human BCL-G regulates secretion of inflammatory chemokines but is dispensable for induction of apoptosis by IFN-γ and TNF-α in intestinal epithelial cells
Source: Cell Death Dis. 2020 Jan 27;11(1):68. doi: 10.1038/s41419-020-2263-0 (PMC6985252; doi:10.1038/s41419-020-2263-0)

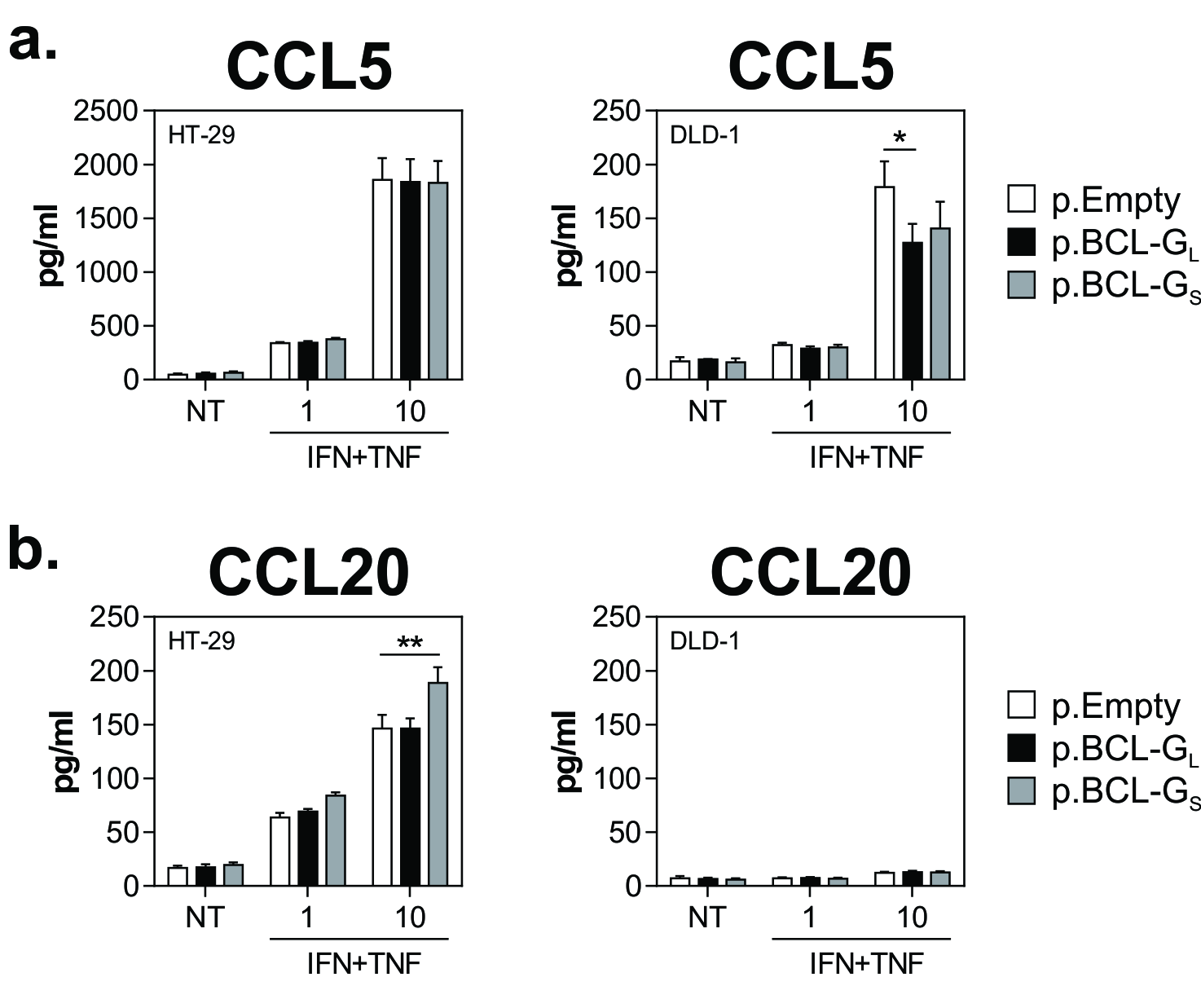

Supplement: Supplementary file 2 — Suppl Figure 1 [file 41419_2020_2263_MOESM2_ESM.tif]
